# Fluorescence Spectroscopic Analysis of ppGpp Binding to cAMP Receptor Protein and Histone-Like Nucleoid Structuring Protein

**DOI:** 10.3390/ijms22157871

**Published:** 2021-07-23

**Authors:** Taner Duysak, Thanh Tuyen Tran, Aqeel Rana Afzal, Che-Hun Jung

**Affiliations:** 1 Department of Molecular Medicine, Chonnam National University, Gwangju 61186, Korea; trduysak@hotmail.com (T.D.); nucuoithienthan2812@gmail.com (T.T.T.); 2 Department of Medical Science, Chonnam National University, Gwangju 61186, Korea; aqeelrana41@gmail.com; 3 Department of Chemistry, Chonnam National University, Gwangju 61186, Korea

**Keywords:** cyclic AMP receptor protein, CRP, histone-like nucleoid structuring protein, H-NS, ppGpp

## Abstract

The cyclic AMP receptor protein (CRP) is one of the best-known transcription factors, regulating about 400 genes. The histone-like nucleoid structuring protein (H-NS) is one of the nucleoid-forming proteins and is responsible for DNA packaging and gene repression in prokaryotes. In this study, the binding of ppGpp to CRP and H-NS was determined by fluorescence spectroscopy. CRP from *Escherichia coli* exhibited intrinsic fluorescence at 341 nm when excited at 280 nm. The fluorescence intensity decreased in the presence of ppGpp. The dissociation constant of 35 ± 3 µM suggests that ppGpp binds to CRP with a similar affinity to cAMP. H-NS also shows intrinsic fluorescence at 329 nm. The fluorescence intensity was decreased by various ligands and the calculated dissociation constant for ppGpp was 80 ± 11 µM, which suggests that the binding site was occupied fully by ppGpp under starvation conditions. This study suggests the modulatory effects of ppGpp in gene expression regulated by CRP and H-NS. The method described here may be applicable to many other proteins.

## 1. Introduction

Since the discovery of cAMP by Sutherland and Rall (1958), molecules in combination with cAMP receptor protein (CRP) and adenylate cyclase have served as the model for signal transduction pathways [1]. A CRP dimer binds two cAMPs with an anti-conformation and two additional cAMPs with a syn-conformation (with a substantially lower affinity). The anti-cAMP binding is responsible for the global allosteric transition of CRP [2,3]. In *Escherichia coli*, CRP (also referred to as catabolite gene activator protein, CAP) regulates the transcription of about 400 genes [4,5]. The cellular concentrations of cAMP in an *Escherichia coli* strain have been reported to vary between 1.5 and 5 µM in the presence of glycerol, and 0.4 and 1.5 µM in the presence of glucose [6,7]. The binding of cAMP to CRP shows negative cooperativity under a low ionic strength, although this is still debated. The dissociation constants for the first cAMP binding to CRP have been reported as 25 and 11 µM by equilibrium dialysis using radioactive cAMP [8,9]. We describe a simple method to determine the binding affinity of ligands to proteins. Since the binding of cAMP to CRP has been studied extensively, previous studies using radioactive ligands may serve as a reference to corroborate the method described in this study. 

At least 12 nucleoid-associated proteins, which play a role in DNA packaging such as eukaryotic histone proteins have been described in *Escherichia coli* [10]. Among them, histone-like nucleoid structuring protein (H-NS) is involved in the biogenesis of flagella; H-NS mutation reduces the transcription of flhD and fliA genes, and thereby results in the complete loss of flagella [11]. The expression of approximately 5% of the genes is altered in the H-NS mutant strain. In most cases, H-NS appears to repress gene expression [12]. Transcription of the H-NS gene is down-regulated by ppGpp, the signaling molecule in bacterial stringent responses [13]. H-NS is an abundant protein, about 20,000 copies of which are present in each cell [14,15]. The binding effect of abundant proteins to signaling molecules may have a more significant impact in cells by sequestering a large portion of signal molecules.

The stringent response is mediated by guanosine tetraphosphate (ppGpp) and guanosine pentaphosphate (pppGpp) in response to nutrient (phosphate, iron, carbon, and fatty acid) deprivation [16,17]. (p)ppGpp also serves as a global regulator in replication, transcription, and translation, including the expression of about 1400 genes [18,19,20,21]. Despite its importance in bacterial physiology and gene regulation and nearly half a century of study, the mechanisms concerning how ppGpp acts on stringent responses remains incompletely understood since the full set of effector proteins is unknown. Recently, CRP has been identified as a ppGpp binding protein in a study utilizing a photo-crosslinking ppGpp analog [22]. H-NS is also captured to a ppGpp-immobilized resin (unpublished data). Nevertheless, the binding of ppGpp to CRP and H-NS has yet to be reported.

In this study, ppGpp binding to CRP and H-NS was determined by fluorescence spectroscopy, while cAMP-binding to CRP was used as a reference. The dissociation constant of cAMP to CRP was similar to those reported previously, implying that the method described here is valid. We report here for the first time that CRP and H-NS bind to ppGpp with ‘high’ affinity, suggesting that their binding may play a role in the regulation of gene expression in *Escherichia coli*.

## 2. Results

### 2.1. Cloning, Over-Expression, and Purification of CRP and H-NS from E. coli

The CRP gene was cloned into *E. coli* BL21 (DE3). CRP and H-NS were over-expressed and isolated as described in the Materials and Methods section. In SDS-PAGE, the isolated CRP and H-NS migrated as single bands with molecular masses around 24 kDa and 16 kDa, respectively (data not shown). CRP was eluted as a single peak in a size exclusion chromatography and the molecular mass was determined as 39–42 kDa, suggesting that the isolated CRP was a dimer, as expected (data not shown).

### 2.2. Binding of ppGpp and cAMP to CRP

**Figure 1:**
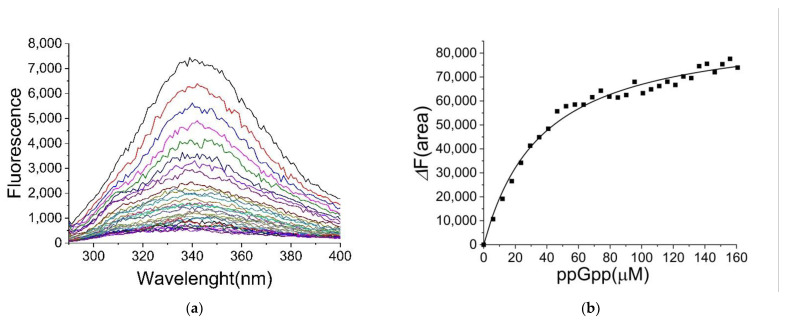
Binding of ppGpp to CRP determined by fluorescence spectroscopy. (**a**) The fluorescence Spectra of CRP in the presence of ppGpp. Following excitation at 280 nm, the emission spectra of CRP (2 µM) were recorded at 25 °C. (**b**) The fluorescence change (ΔF(area)) against ppGpp concentrations. Instead of using the fluorescence change at a fixed wavelength, the area below the spectra between 334 and 348 nm was used, and the area decrease (ΔF(area)) upon addition of ppGpp was utilized to calculate the extent of fluorescence quenching. The *K_D_* value for ppGpp-binding to CRP was calculated as 35 ± 3 µM.

*E. coli* CRP showed an intrinsic fluorescence when excited at 280 nm. The fluorescence intensity was decreased by the presence of ppGpp (Figure 1a). The dissociation constant of ppGpp binding to CRP was calculated as 35 ± 3 μM based on the changes in the areas below the spectra (Figure 1b). The binding of various ligands (including cAMP) was also investigated (Supplementary Figure S1) and the results are summarized in Table 1. The dissociation constant for cAMP to CRP was determined as 22 ± 3 μM, which was consistent with previous reports [8,9,23]. The calculated dissociation constants for cGMP and ATP were 17 ± 1 and 33 ± 4 μM, respectively, suggesting that ppGpp, cGMP, and ATP bound to CRP with a similar affinity as cAMP. Based on equilibrium dialysis utilizing radioactive ligands, the binding of cGMP and cAMP to CRP (with a similar affinity) was reported previously [8], suggesting that the method described here was valid. The current method was much simpler and not limited by the availability of radioactive ligands. ATP binding to CRP with a high affinity was an unexpected observation.

### 2.3. The Binding Site for ppGpp on CRP

X-ray crystallography showed significant structural changes in CRP upon binding with cAMP and cGMP [2,24]. Since the crystal structure of ppGpp-bound CRP was not available, a docking simulation for ppGpp binding to CRP was performed. Since the protein structures served as templates and remained constant during docking simulation, the structural changes upon binding of ligand could not be simulated. In this study, two crystal structures of the ligand-bound forms, including cAMP and cGMP-bound forms, were used and compared. It was reasoned that one of the structures might be a better fit with ppGpp and resemble the CRP structure following ppGpp binding. Five amino acid residues including Q80, E81, R82, S83, and A84, composing of the binding site for cAMP and cGMP, were selected as the ‘binding pocket residues’. The energy minimization results from an individual selection of the amino acids are summarized in Table 2, as the docking software provided the results of simulations. The software predicted that ppGpp might form a more stable complex with the cGMP-bound structure of CRP in general, which suggested that ppGpp, like cGMP, might down-regulate CRP activity.

**Figure 2:**
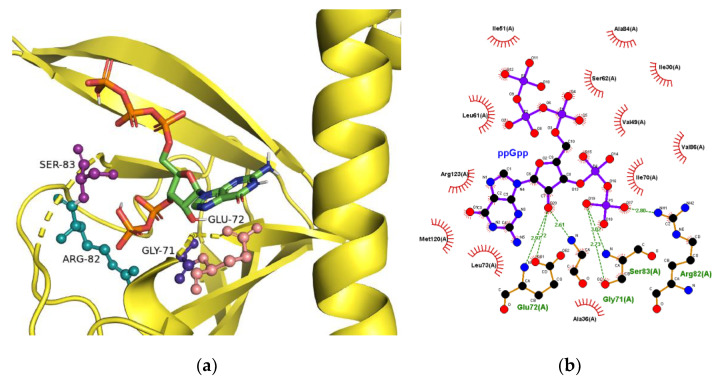
Interaction of ppGpp with CRP. (**a**) The ppGpp-binding site proposed by a molecular modeling study. The interactions between ppGpp and CRP are shown as dotted lines. (**b**) Interacting residues at the binding site. G71, R82, and S83 are commonly interacting amino acid residues in cAMP, cGMP and ppGpp-bound CRP.

The proposed three-dimensional structure and the ligand-protein interactions, when S83 was designated as the binding pocket residue, are shown in Figure 2. The comparison of cAMP- and cGMP-bound CRP structures showed that G71, R82, and S83 interacted with ribose and phosphate moieties of the ligands [2,24]. In contrast, the adenine base interacted with S83 with an anti-conformation while guanine base with T27 with a syn-conformation. As shown in Figure 2b, G71, R82, and S83 were proposed to participate again in the interaction with ppGpp. The guanine base of ppGpp, like cGMP, was present as a syn-conformer in a hydrophobic pocket.

### 2.4. Binding of ppGpp and cAMP to H-NS

**Figure 3:**
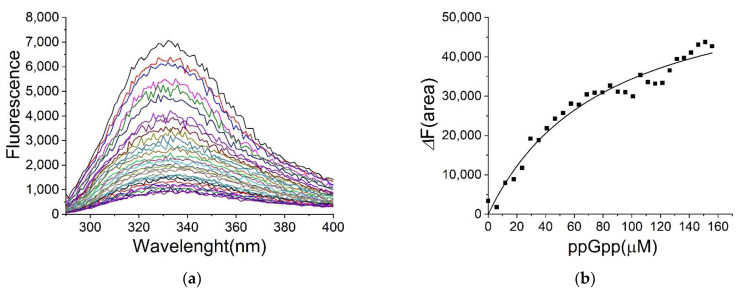
Determination of the dissociation constant *K_D_* for ppGpp on H-NS by fluorescence spectrometry. (**a**) The emission spectra of H-NS in the presence of ppGpp. While H-NS (10µM) was excited at 280 nm, the emission spectra in the presence of various concentrations of ppGpp were recorded at 25 °C. (**b**) The difference in fluorescence (ΔF(area)) between 322 and 336 nm against ppGpp concentrations. The *K_D_* for ppGpp was calculated as 80 ± 11 µM.

*E. coli* H-NS also showed an intrinsic fluorescence when excited at 280 nm, and the fluorescence intensity decreased in the presence of ppGpp (Figure 3a). The sole tryptophan residue at the position 109 might be responsible for the fluorescence of H-NS. The fluorescence changes were again utilized in determining the dissociation constants (Figure 3b). As shown in Table 2, the dissociation constants of ppGpp and cAMP to H-NS were 80 ± 11 μM and 270 ± 40 μM, respectively. ATP binding did not show a saturation curve, indicating a high *K_D_* value (Table 3 and Supplementary Figure S2). We compared in vitro transcription in the presence and absence of ppGpp and found no changes with ppGpp (data not shown). The effect of ppGpp binding with H-NS is not clear at this point.

### 2.5. The Binding Site for ppGpp on H-NS

*E. coli* H-NS is a protein with 137 amino acids. The three-dimensional structure of H-NS full sequence from any sources has not been reported yet. For *E. coli*, the NMR structures of fragment 2–58 (1LR1), fragment 2–47 (1NI8), and fragment 1–47 complexed with Hha (2MW2) are available at the protein data bank. A structure of C-terminal fragment 91–137 (1HNR) is also presented. The fragment 91–137 is monomeric, while all of the other fragments are described as dimers. The three-dimensional structures of three N-terminal fragments are very different, suggesting a flexible nature of H-NS. The docking with GalaxyWeb server did not provide the binding pocket when the above four structures were used as templates.

**Figure 4:**
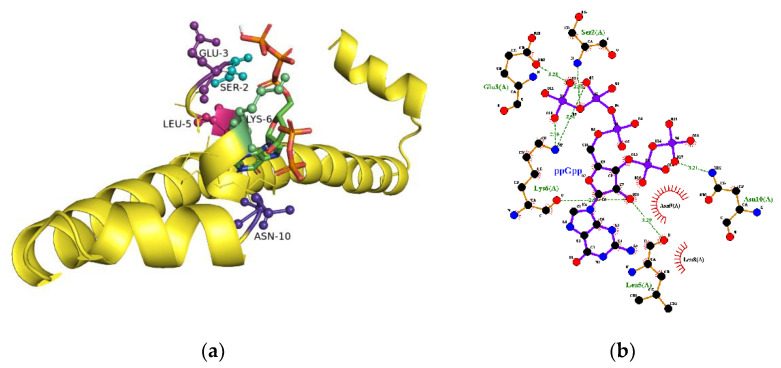
Interaction of ppGpp with N-NS. (**a**) The ppGpp binding site proposed by a molecular modeling study. The interactions between ppGpp and H-NS are shown as dotted lines. (**b**) Interacting residues at the binding site. S2, E3, K6, and N10 interact with phosphate groups of ppGpp.

H-NS from *Salmonella typhimurium* is 95% identical to that from *E. coli*. The structure from an x-ray crystallographic study of H-NS fragment 1–83 (3NR7) is available as a monomeric peptide. Only two amino acids, L79 and V82, are different in the fragment and the docking with GalaxyWeb server suggests the binding site for ppGpp. As shown in Figure 4, the amino acids at the N-terminal of H-NS are proposed to interact with ppGpp. Since the structure of the whole H-NS is not available, the relationship between binding site and the fluorophore W109 is unknown. The structural flexibility of H-NS suggests that H-NS may undergo a structural transition in the presence of ppGpp.

## 3. Discussion

Two tryptophan residues at the positions 14 and 86 of CRP contribute to the intrinsic fluorescence. Wasylewski et al. suggested the predominance of fluorescence by W14 [25], which might attenuate the complexity due to the presence of multiple fluorophores. Takahashi et al. reported that cGMP bound to CRP competitively with cAMP had a similar affinity, but the effect of their binding on CRP deferred. The cGMP binding properties remained unchanged in the presence of double-stranded deoxyribonucleic acid (dsDNA), while cAMP bound more efficiently to dsDNA-bound CRP [8]. This is the first report showing ppGpp binding to CRP, suggesting that the effect of ppGpp binding to CRP may be similar to cGMP-binding and that the gene expression by CRP may be further modulated by ppGpp binding to CRP under starvation conditions.

Surprisingly, ATP bound to CRP with a similar affinity as cAMP. In glucose-fed, exponentially growing *Escherichia coli*, the cellular concentrations of ATP and cAMP were 9600 μM and 35 μM, respectively [26]. Since over-estimation of cAMP is well-documented [7], it can be assumed that the difference in ATP and cAMP concentrations may be substantially higher. To overcome the huge difference in concentration, the binding affinity for cAMP must increase significantly in the presence of DNA and RNA polymerase. Otherwise, cAMP will never have a chance to compete with ATP.

Johansson et al. reported that H-NS stimulated stringently controlled bacterial genes [27]. *E. coli* lacking both H-NS and its paralog, StpA, showed a lower expression of CRP and reduced growth. The growth rate was restored in the mutant with impaired ppGpp synthesis; overexpression of CRP partially compensated the reduced growth. Although their study links CRP with the (p)ppGpp regulons in *E. coli*, this study, demonstrating direct interactions of ppGpp to H-NS and CRP, seems to be unrelated to their studies. The cellular concentrations of ppGpp reaches millimolar levels under starvation conditions [28,29]. Therefore, the cellular ppGpp concentration under starvation conditions may be 10–20 times higher than the dissociation constant (80 ± 11 μM), and the binding site on H-NS may be fully occupied by ppGpp under physiological conditions. Based on the cellular concentration of ppGpp and the dissociation constants, the binding affinities of ppGpp to H-NS and CRP may be considered as ‘strong’ ones.

CRP is a dimeric protein and each CRP monomer binds with 2 molecules of cAMP. The first binding of cAMP, with an approximate *K_D_* of 20 μM, activates CRP. The second binding with syn-conformation of cAMP, however, down-regulates CRP. The *K_D_* for the second site is around 2 mM [30]. The binding site of cAMP on H-NS, with eight-fold higher affinity than the second cAMP site on CRP, appears to be occupied fully only when the second binding site on CRP is occupied by cAMP.

Recent developments in proteomics report larger numbers of proteins for analysis. For example, one study utilizing a photo-crosslinking ppGpp analog has captured 299 proteins as ppGpp binding proteins [22]. To analyze the binding affinities of those proteins for ppGpp, a rapid and reliable method may be required. Fluorescence spectroscopic analysis, used to determine ligand binding, has been introduced over 30 years ago. However, intrinsic fluorescence of proteins may be considered too weak and its utilization for ligand binding study has not been explored much. This study demonstrates that the fluorescence intensities of CRP and H-NS are significant in that the binding of various ligands can be measured without the labelling of proteins with fluorophores. In addition to the new findings of ppGpp binding to CRP and H-NS, this provides a simple and label-free method to screen various protein-ligand binding.

## 4. Materials and Methods

Imidazole, ATP, GTP, GDP, and cAMP were purchased from Sigma-Aldrich Korea, Ltd. (Seoul, Korea). *E. coli* DH5α and BL21 (DE3) were obtained from Promega Korea Ltd. (Daejeon, Korea). The restriction enzymes were ordered from New England Biolabs (Ipswich, MA, USA). Ni-NTA and desalting columns were purchased from GE Healthcare Korea (Seoul, Korea). PCR primers were obtained from Bioneer corp. (Daejeon, Korea). ppGpp was synthesized and isolated as described previously [31,32].

### 4.1. Cloning of CRP from E. coli

A genomic DNA was prepared from *E. coli* K12 MG1655. The CRP gene encoding cAMP receptor protein was amplified by polymerase chain reaction (PCR), using these primers; 5′-GTTAATTACACTCAATCGAGTGAGTAATCC-3′ (forward) and 5′-CAATTAATGTGAGCTCACTCATTAGG-3′ (reverse). The PCR product and pET21a(+) vector were treated with *Nde*I and *Xho*I, and ligated. The plasmid construct was transformed to DH5α and subsequently to BL21 (DE3).

### 4.2. Over-Expression and Isolation of H-NS and CRP

The strain over-expressing H-NS was obtained from Dr. Hyon E. Choy at Chonnam National University, and was originally constructed at Dr. Bremer’s laboratory in FRG. The transformed BL21 (DE3) strains were grown at 37 °C in LB media containing 50 μg/mL of ampicillin until OD_600_ reached 0.5–0.6. IPTG (0.2 mM) was added for induction, and the cells were further incubated for 4 h at 37 °C and harvested by centrifugation at 4000× *g* for 30 min. For isolating H-NS, the cells were re-suspended in 50 mM Tris-HCl buffer, pH 8.0, containing 1 mM PMSF, and disrupted by sonication. The soluble fraction was applied to a Ni-NTA column pre-equilibrated with 50 mM Tris-HCl, pH 8.0 containing 10 mM imidazole and 300 mM NaCl. H-NS was eluted with a linear gradient (10–500 mM) of imidazole gradient in the same buffer. Fractions containing H-NS were pooled and applied to a desalting column pre-equilibrated with 50 mM Tris-HCl, pH 8.0 for buffer exchange. For isolating CRP, the cells were suspended in 50 mM KH_2_PO_4_ buffer, pH 6.0, containing 1 mM PMSF, and disrupted by sonication. The supernatant was applied to the Ni-NTA column pre-equilibrated with 50 mM KH_2_PO_4_, pH 6.0, containing 0.3 M NaCl and 20 mM imidazole. CRP was eluted with a linear gradient (20–800 mM) of imidazole in the same buffer. The fractions containing CRP were pooled and applied to a desalting column to exchange buffer with 50 mM MOPS, pH 7.5. The purities of H-NS and CRP were assessed via SDS-PAGE. The protein concentrations were determined by measuring absorbance at 280 nm [33].

### 4.3. Determination of Ligand Binding by Fluorescence Spectroscopy

Fluorescence intensities were measured at 25 °C using a F-4500 Fluorescence Spectrophotometer (Hitachi corporation). H-NS and CRP in 50 mM MOPS buffer, pH 7.5 were excited at 280 nm and the emission spectra were recorded from 290 to 400 nm under various ligand concentrations. The dissociation constant, *K_D_*, was calculated using the equation, ΔF/ΔF_M_ = (([L] + [P] + *K_D_*) − (([L] + [P] + *K_D_*)^2^ − 4·[L]·[P])^0.5^)/2·[P], where ΔF, ΔF_M_, [P], and [L] represent fluorescence change, the maximal fluorescence change, total protein concentration, and total ligand concentration, respectively [34,35]. The area below the spectra in the 15 nm ranges was used to calculate ΔF in this study. The corrections for the inner filter effect, introduced by the ligands, was performed using the formula, F_cor_ = F × 10^(P + ΔA)/2^, where F and F_cor_ denote fluorescence intensity before and after the correction, whereas P and ΔA indicate the initial sample absorption at the excitation wavelength and the change in absorption introduced by ligands, respectively [36].

### 4.4. Computational Prediction of ppGpp-Binding to CRP

The two structures (1g6n and 1n9i) of *E. coli* CRP, occurring as cAMP- or cGMP-bound forms, were used for docking simulation. The docking models were prepared by removing the corresponding ligands from the structures. The prepared ppGpp (CID: 135398636) and the CRP models were simulated for the protein-ligand docking. The docking, energy minimization, and scoring of the results were performed using GalaxyWEB server as described previously [37,38,39] The generated structures were visualized using PyMol [40,41].

## Figures and Tables

**Table 1:** The *K_D_*’s for nucleotide ligands on CRP.

Ligand	*K_D_* (µM)
ppGpp	35 ± 3
GDP	62 ± 4
GTP	35 ± 3
cGMP	17 ± 1
cAMP	22 ± 3
ATP	33 ± 4
cAMP ^a^	25 and 11 ^a^

^a^, reported previously by equilibrium dialysis [8,9].

**Table 2:** The docking parameters.

Binding Pocket Residue	1g6n (cAMP Form)	1n9i (cGMP Form)
Q80	−9.90	−10.53
E81	−7.07	−10.43
R82	−9.42	−13.48
S83	−10.05	−14.01
A84	−10.33	−12.60

**Table 3:** The *K_D_*’s for nucleotide ligands on H-NS.

Ligand	*K_D_* (µM)
ppGpp	80 ± 11
GDP	72 ± 6
GTP	120 ± 25
cAMP	270 ± 40
ATP	NS ^1^

^1^ NS, not saturated.

## Data Availability

The data shown in this study are presented in the article. The data presented in this study are also available on request from the corresponding authors.

## Supplementary Materials

The following are available online at https://www.mdpi.com/article/10.3390/ijms22157871/s1.

## References

[1] Sutherland E., Rall T. (1958). Fractionation and characterization of a cyclic adenine ribonucleotide formed by tissue particles. J. Biol. Chem..

[2] Passner J., Schultz S., Steitz T. (2000). Modeling the cAMP-induced Allosteric Transition Using the Crystal Structure of CAP-cAMP at 2.1Å Resolution. J. Mol. Biol..

[3] Parkinson G., Wilson C., Gunasekera A., Ebright Y.W., Ebright R., Berman H.M. (1996). Structure of the CAP-DNA Complex at 2.5 Å Resolution: A Complete Picture of the Protein-DNA Interface. J. Mol. Biol..

[4] Hollands K., Busby S.J., Lloyd G.S. (2007). New targets for the cyclic AMP receptor protein in theEscherichia coliK-12 genome. FEMS Microbiol. Lett..

[5] Shimada T., Fujita N., Yamamoto K., Ishihama A. (2011). Novel Roles of cAMP Receptor Protein (CRP) in Regulation of Transport and Metabolism of Carbon Sources. PLoS ONE.

[6] Epstein W., Rothman-Denes L.B., Hesse J. (1975). Adenosine 3’:5’-cyclic monophosphate as mediator of catabolite repression in *Escherichia coli*. Proc. Natl. Acad. Sci. USA.

[7] Pastan I., Adhya S. (1976). Cyclic adenosine 5′-monophosphate in *Escherichia coli*. Bacteriol. Rev..

[8] Takahashi M., Blazy B., Baudras A. (1980). An equilibrium study of the cooperative binding of adenosine cyclic 3′,5′-monophosphate and guanosine cyclic 3′,5′-monophosphate to the adenosine cyclic 3′,5′-monophosphate receptor protein from *Escherichia coli*. Biochemistry.

[9] Heyduk T., Lee J.C. (1989). *Escherichia coli* cAMP receptor protein: Evidence for three protein conformational states with different promoter binding affinities. Biochemistry.

[10] Azam T.A., Ishihama A. (1999). Twelve Species of the Nucleoid-associated Protein from *Escherichia coli*. J. Biol. Chem..

[11] Bertin P., Terao E., Lee E.H., Lejeune P., Colson C., Danchin A., Collatz E. (1994). The H-NS protein is involved in the biogenesis of flagella in *Escherichia coli*. J. Bacteriol..

[12] Hommais F., Krin E., Laurent-Winter C., Soutourina O., Malpertuy A., Le Caer J.-P., Danchin A., Bertin P. (2001). Large-scale monitoring of pleiotropic regulation of gene expression by the prokaryotic nucleoid-associated protein, H-NS. Mol. Microbiol..

[13] Brandi A., Giangrossi M., Fabbretti A., Falconi M. (2020). The *hns* Gene of *Escherichia coli* Is Transcriptionally Down-Regulated by (p)ppGpp. Microorganism.

[14] Lu P., Vogel C., Wang R., Yao X., Marcotte E.M. (2007). Absolute protein expression profiling estimates the relative contributions of transcriptional and translational regulation. Nat. Biotechnol..

[15] Arikea L., Valgepeaa K., Peilc L., Nahkua R., Adamberga K., Vilua R. (2012). Comparison and applications of label-free absolute proteome quantification methods on *Escherichia coli*. J. Proteom..

[16] Cashel M., Gentry D.R., Hernandez V.H., Vinella D., Neidhardt F.C. (1996). The stringent response. Escherichia coli and Salmonella: Cellular and Molecular Biology.

[17] Dalebroux Z.D., Svensson S.L., Gaynor E.C., Swanson M.S. (2010). ppGpp Conjures Bacterial Virulence. Microbiol. Mol. Biol. Rev..

[18] Magnusson L.U., Farewell A., Nyström T. (2005). ppGpp: A global regulator in *Escherichia coli*. Trends Microbiol..

[19] Wang J., Sanders G.M., Grossman A.D. (2007). Nutritional Control of Elongation of DNA Replication by (p)ppGpp. Cell.

[20] Srivatsan A., Wang J.D. (2008). Control of bacterial transcription, translation and replication by (p)ppGpp. Curr. Opin. Microbiol..

[21] Traxler M., Summers S.M., Nguyen H.-T., Zacharia V.M., Hightower G.A., Smith J.T., Conway T. (2008). The global, ppGpp-mediated stringent response to amino acid starvation in *Escherichia coli*. Mol. Microbiol..

[22] Wang B., Dai P., Ding D., Del Rosario A., Grant R.A., Pentelute B.L., Laub M.T. (2019). Author Correction: Affinity-based capture and identification of protein effectors of the growth regulator ppGpp. Nat. Chem. Biol..

[23] Harman J.G. (2001). Allosteric regulation of the cAMP receptor protein. Biochim. Biophys. Acta.

[24] Seok S.-H., Im H., Won H.-S., Seo M.-D., Lee Y.-S., Yoon H.-J., Cha M.-J., Park J.-Y., Lee B.-J. (2014). Structures of inactive CRP species reveal the atomic details of the allosteric transition that discriminates cyclic nucleotide second messengers. Acta Crystallogr. Sect. D Biol. Crystallogr..

[25] Wasylewski M., Małecki J., Wasylewski Z. (1995). Fluorescence study of *Escherichia coli* cyclic AMP receptor protein. J. Protein Chem..

[26] Bennett B.D., Kimball E.H., Gao M., Osterhout R., Van Dien S.J., Rabinowitz J.D. (2009). Absolute metabolite concentrations and implied enzyme active site occupancy in Escherichia coli. Nat. Chem. Biol..

[27] Johansson J., Balsalobre C., Wang S.-Y., Urbonaviciene J., Jin D.J., Sondén B., Uhlin B.E. (2000). Nucleoid Proteins Stimulate Stringently Controlled Bacterial Promoters: A Link between the cAMP-CRP and the (p)ppGpp Regulons in *Escherichia coli*. Cell.

[28] Cashel M. (1969). The control of ribonucleic acid synthesis in *Escherichia coli*. IV. Relevance of unusual phosphorylated compounds from amino acid starved stringent strains. J. Biol. Chem..

[29] Haseltine W.A., Block R. (1973). Synthesis of guanosine tetra- and pentaphosphate requires the presence of a codon-specific, uncharged transfer ribonucleic acid in the acceptor site of ribosomes. Proc. Natl. Acad. Sci. USA.

[30] Malecki J., Polit A., Wasylewski Z. (2000). Kinetic studies of cAMP-induced allosteric changes in cyclic AMP receptor protein from *Escherichia coli*. J. Biol. Chem..

[31] Krohn M., Wagner R. (1995). Procedure for the Rapid Preparation of Guanosine Tetraphosphate (ppGpp) from *Escherichia coli* Ribosomes. Anal. Biochem..

[32] Hoang H.N., Tran T.T., Jung C. (2020). The Activation of Glycerol Dehydrogenase from *Escherichia coli* by ppGpp. Bull. Korean Chem. Soc..

[33] Gill S.C., Von Hippel P.H. (1989). Calculation of protein extinction coefficients from amino acid sequence data. Anal. Biochem..

[34] Clark A.R., Engel P.C. (1996). Analysis of ligand binding by enzymes. Enzymology Labfax.

[35] Duysak T., Afzal A.R., Jung C.-H. (2021). Determination of glutathione-binding to proteins by fluorescence spectroscopy. Biochem. Biophys. Res. Commun..

[36] Lakowicz J.R. (1999). Principles of Fluorescence Spectroscopy.

[37] Shin W.H., Heo L., Lee J., Ko J., Seok C., Lee J. (2011). LigDockCSA: Protein-ligand docking using conformational space annealing. J. Comput. Chem..

[38] Shin W.H., Kim J.K., Kim D.S., Seok C. (2013). GalaxyDock2: Protein-ligand docking using beta-complex and global optimization. J. Comput. Chem..

[39] Baek M., Shin W.H., Chung H.W., Seok C. (2017). GalaxyDock BP2 score: A hybrid scoring function for accurate protein-ligand docking. J. Comput. Aided Mol. Des..

[40] (2019). The PyMOL Molecular Graphics System.

[41] Wallace A.C., Laskowski R.A., Thornton J.M. (1995). LIGPLOT: A program to generate schematic diagrams of protein-ligand in-teractions. Protein Eng..

