# Supplementary material for: Fluorescence Spectroscopic Analysis of ppGpp Binding to cAMP Receptor Protein and Histone-Like Nucleoid Structuring Protein
Source: Int J Mol Sci. 2021 Jul 23;22(15):7871. doi: 10.3390/ijms22157871 (PMC8346002; doi:10.3390/ijms22157871)
Supplement: Supplementary file 1 [file ijms-22-07871-s001.zip › ijms-1265761-supplementary.pdf]

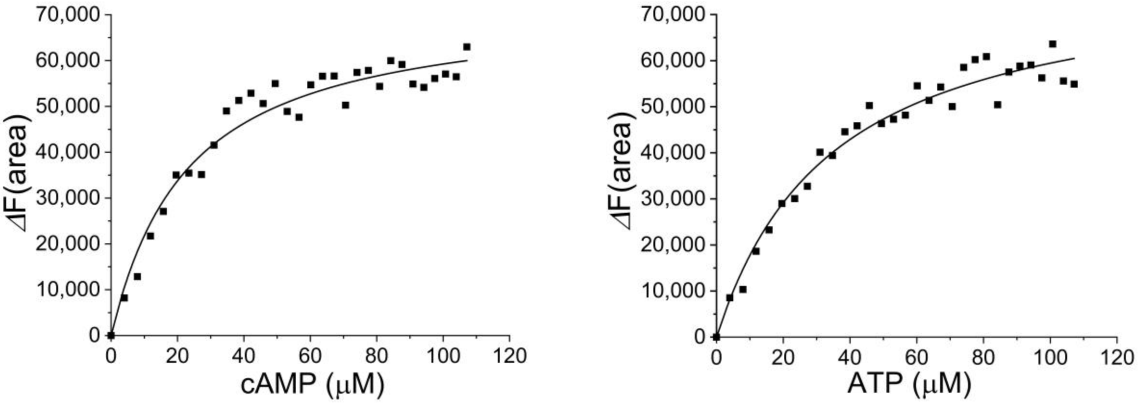

(**a**) (**b**)

Supplementary Figure S1. Determination of the dissociation constant K_D's for cAMP and ATP to CRP by fluorescence spectroscopy. (a) The fluorescence change (ΔF(area)) between 334 and 348 nm against cAMP concentrations. The emission spectra of CRP (2 μM) were recorded at 25 ℃. The K_D values for cAMP-binding to CRP was calculated as 22 ± 3 μM. (b) The fluorescence change (ΔF(area)) between 334 and 348 nm against ATP concentrations. The K_D values for ATP-binding to CRP was 33 ± 4 μM.

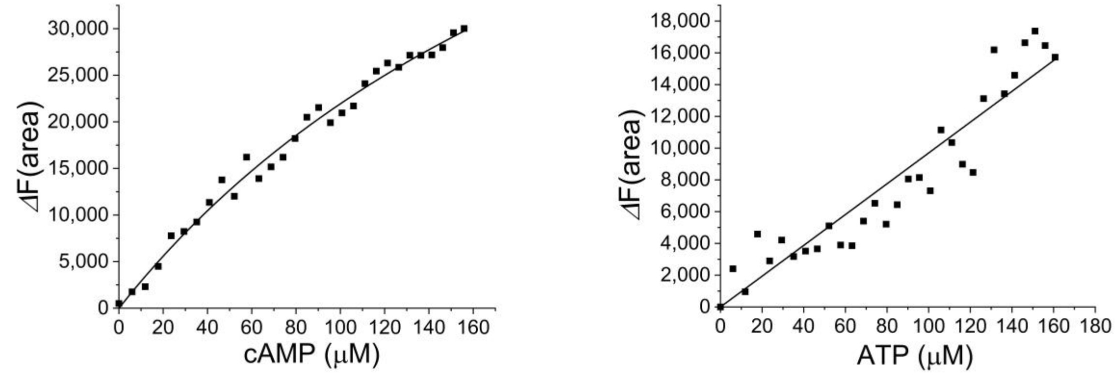

(**a**) (**b**)

Supplementary Figure S2. The dissociation constant K_D's for cAMP and ATP to H-NS determined by fluorescence spectroscopy. (a) The fluorescence change (ΔF(area)) between 322 and 336 nm against cAMP concentrations. The emission spectra of N-NS (10 μM) were recorded at 25 ℃. The K_D values for cAMP-binding to H-NS was calculated as 270 ± 40 μM. (b) The fluorescence change (ΔF(area)) between 322 and 336 nm against ATP concentrations. ATP-binding does not show a saturation curve, suggesting a high K_D value.
